# Characterization of the Psychological, Physiological and EEG Profile of Acute Betel Quid Intoxication in Naïve Subjects

**DOI:** 10.1371/journal.pone.0023874

**Published:** 2011-08-31

**Authors:** Peter G. Osborne, Tung-Shan Chou, Tsu-Wang Shen

**Affiliations:** 1 Baongong Agri-Science Center, Taidong, Taiwan; 2 Department of Counseling and Clinical Psychology, National DongHwa University, Hualien, Taiwan; 3 Department of Medical Informatics, TzuChi University, Hualien, Taiwan; Centre for Addiction and Mental Health, Canada

## Abstract

Betel quid use and abuse is wide spread in Asia but the physiological basis of intoxication and addiction are unknown. In subjects naïve to the habit of betel quid intoxication, the psychological and physiological profile of intoxication has never been reported. We compared the effect of chewing gum or chewing betel quid, and subsequent betel quid intoxication, on psychological assessment, prospective time interval estimation, numerical and character digit span, computerized 2 choice tests and mental tasks such as reading and mathematics with concurrent monitoring of ECG, EEG and face temperature in healthy, non-sleep deprived, male subjects naïve to the habit of chewing betel quid. Betel quid intoxication, dose dependently induced tachycardia (max 30 bpm) and elevated face temperature (0.7°C) (P<0.001) above the effects observed in response to chewing gum (max 12 bpm and 0.3°C) in 12 subjects. Gross behavioral indices of working memory such as numerical or character digit span in 8 subjects, or simple visual-motor performance such as reaction speed or accuracy in a two choice scenario in 8 subjects were not affected by betel quid intoxication. Betel quid intoxication strongly influenced the psychological aspects of perception such as slowing of the prospective perception of passage of a 1 minute time interval in 8 subjects (P<0.05) and perceived increased arousal (P<0.01) and perceived decreased ability to think (P<0.05) in 31 subjects. The EEG spectral profile recorded from mental states associated with open and closed eyes, and mental tasks such as reading and eyes closed mental arithmetic were significantly modified (P<0.05) relative to chewing gum by betel quid intoxication in 10 subjects. The prevalence of betel quid consumption across a range of social and work settings warrants greater investigation of this widespread but largely under researched drug.

## Introduction

The chewing of the nut of the *Areca catechu* palm together with a strong base, frequently a form of calcium hydroxide, and invariably accompanied with the leaf/inflorescence of *Piper betle*, hereafter referred to collectively as betel quid, is estimated to be practiced by 200–600 million people inhabiting Asian regions from Southern India to Papua New Guinea [1], [2], [3]. Gender differences are apparent in the prevalence of betel quid use between Asian cultures [4]. After alcohol, tobacco and caffeine, betel quid is probably the most common drug of addiction in the world but is little known outside Asia [3]. In Taiwan, the demographics of chewing betel quid is strongly associated with males of lower socioeconomic groups and up to 2.5 million people of a population of 25 million people are estimated to be habitual chewers [1]. The neuro-chemical basis of betel quid addiction is unknown and there are no behavioral programs directed at minimizing addiction and chronic use of betel quid.

Chewing betel quid, releases pharmacologically active components that are absorbed into the blood via the mucosal membranes of the mouth and intestine and acts on the brain and periphery to cause physiological effects such as tachycardia, salivation, miosis, tremor and psychological effects that have been variously described as euphoria, drunkenness, restfulness and central nervous system stimulation [5], [1], [2], [3]. The pharmacologically active components of betel quid are derived from both the organic compounds released by chewing, hydrolysation of these compounds in the strongly basic oral environment and also metabolism. The nut of the *Areca catechu* contains at least 1% arecholine [6] and this muscarinic (M1) agonist is considered to be the predominant alkaloid. In addition, the pharmacologically active compounds in the betel quid include GABA uptake inhibitors, guvacine and arecaidine [7], monoamine oxidase A inhibitors [8], acetylcholine esterase inhibitors [9] and metabolites of arecoline and arecaidine with unknown physiological function [10].

Betel quid components exhibit genotoxic activity and may alter the structure of DNA, proteins and lipids [11]. Analysis of Taiwanese hospital records and national health surveys indicate a strong correlation between betel quid chewing and esophageal cancer [12]; oral cancer [11]; obesity [13] and hypertension [14]. A gradual increase of the incidences of oral pathologies associated with betel quid chewing are being reported in European and American clinics as a result of migration of Asian people [15], [16].

The peripheral effects and time course of the effects of betel quid chewing such as tachycardia, hypersalivation [1], [5] and facial blood vessel vasodilatation and skin temperature increase [17] have been documented and are attributed primarily to the cholinergic agonist properties of arecoline. The central nervous system effects of betel quid intoxication on human cognition and behaviour have not been rigorously investigated. It is the general consensus among users that betel quid chewing induces an increase in arousal [1], [2], [3], [5]. This is consistent with the cortical arousing effects of pure muscarinic agonists including arecoline [18], [19] and the results of single electroencephalographic (EEG) study on habitual betel quid chewers [20]. This study demonstrated that betel quid intoxication in eye closed habitual chewers increased spectral power of alpha and beta waves and decrease power of theta wave that were interpreted as indices of increased alertness associated with a calming state [20].

Psychological examination of the effects of betel quid chewing has been limited to two analyses performed more than 15 years ago on habitual betel quid users. The experimental designs of these studies vary slightly, but demonstrate that although considered arousing or alerting, betel quid chewing had no effect on reaction time [5], [21], hand eye co-ordination [5], inconsistently effected visual choice reaction time [5], [21] and no effect upon working memory [5].

These considerations led us to re-examine the effect of betel quid intoxication on subjects naïve to the habit of betel quid intoxication in a balanced experimental design that examined prospective time interval estimation, digit span, visual choice reaction time and accuracy, and EEG during the performance of mental tasks such as reading and mental arithmetic. This knowledge is a necessary first step in characterizing the psychological effects of betel chewing needed for initiating behavioral programs directed at minimizing addiction and chronic use of betel quid.

## Results

### Experiment 1. Prospective time interval estimation

All subjects reported that betel quid elevated heart rate and face temperature by about 2 minutes after the onset of chewing, consistent with the results of experiment 4. Some subjects said they felt momentarily dizzy while chewing but this was resolved at the time of testing which commenced about 1 minute after the subjects had finished chewing. Of the eight subjects tested all stated that betel quid tasted bad, seven stated that betel quid made concentration more difficult. One subject, the oldest subject, stated that he was confident that betel quid helped him focus. This subject performed poorest in memory tests and was atypical in his estimation of prospective time intervals. Table 1 shows that the subjects mean estimate of the passage of 5 seconds was not different after chewing gum, chewing betel quid or not chewing. The subjects mean estimate of the passage of 1 minute was increased after the subject chewed betel quid. Removal of the atypical subject revealed a significant slowing of prospective time interval estimation after chewing betel quid relative to chewing gum or no chewing.

**Table 1:** Effect of chewing treatments on prospective time interval estimation in seconds.

Time Interval Estimated	Chew Gum Mean ± sem (n)	Chew Betel Quid Mean ± sem (n)	Wait 2 minutes, no chewing. Mean ± sem (n)	ANOVA F statistic, P
5 seconds	5.35±0.37 (8)	5.51±0.36 (8)	4.88±0.28 (8)	F(2,23) = 0.95, ns
5 seconds	5.39±0.42 (7)	5.65±0.38 (7)	4.92±0.32 (7)	F(2,20) = 0.92, ns
60 seconds	59.38±1.64 (8)P<0.05	69.40±3.73 (8)	62.35±1.85 (8)	F(2,23) = 4.1, P<0.032
60 seconds	57.43±1.54 (7) P<0.01	71.32±3.7 (7)	60.97±1.43 (7)P<0.05	F(2,20) = 7.2, P<0.005

P = difference from chew betel quid.

### Experiment 2. Working Memory tests

All subjects reported that betel quid elevated heart rate and face temperature by about 2 minutes after the onset of chewing, consistent with the results of experiment 4. Digit span was quantified by recording the longest correct sequence. Digit score was quantified by assigning each correctly recalled sequence a score of 1 and summing the score for each treatment. Table 2 shows that chewing betel quid had an effect not different from chewing gum on ascending or descending numerical digit span or digit score. There was no difference in the digit span or score of random non vowel English letters after chewing gum or betel quid. Combining gum and betel quid treatments the mean maximum digit span of numbers 8.9±0.2 (n = 32 measurements) was significantly longer than the mean maximum digit span of randomly presented non vowel alphabet characters 6.4±0.4 (n = 16 measurements) (t = 9.9, df = 15, P<0.0001).

**Table 2:** Effect of chewing gum or betel quid on digit span.

Test Parameter	Chew Gum Mean ± sem (n)	Chew Betel Quid Mean ± sem (n)	Paired t-test significance
Digit span Ascending numerical	9.3±0.4 (8)	8.8±0.5 (8)	ns
Ascending score	5.9±0.4 (8)	5.5±0.4 (8)	ns
Digit span Descending numerical	8.8±0.4 (8)	9.1±0.5 (8)	ns
Descending score	5.4±0.5 (8)	5.8±0.6 (8)	ns
Digit span Random character	6.4±0.5 (8)	6.5±0.6 (8)	ns
Character score	4.3±0.6 (8)	4.4±0.6 (8)	ns
Degree of difficulty	2	8	P<0.01

### Experiment 3. Two choice reaction test

All subjects stated that betel quid made their face hot and their heart beat faster after chewing betel quid. Of the eight subjects tested four stated that betel quid tasted bad, all stated that betel quid made concentration more difficult.

Table 3 shows that despite subjects estimating that chewing betel quid made them feel more excited or alert and concentration was more difficult, no difference was found in the reaction times or percent of errors between chewing betel quid or chewing gum.

**Table 3:** Effect of chewing gum or betel quid on 2 choice reaction task.

Test parameter	Chew Gum Mean ± sem (n)	Chew Betel Quid Mean ± sem (n)	Paired t-test significance
Reaction time (mS)	2849±252 (8)	2759±277 (8)	ns
Errors (%)	0.6±0.2 (8)	0.5±0.2 (8)	ns
Taste. Bad = 1/Good = 10	6.4±0.8 (8)	5.2±0.8 (8)	ns
Concentration Easy = 1/Difficult = 10	3.0±0.4 (8)	6.4±0.6 (8)	P<0.001
Restful = 1/Excited = 10	2.5±0.2 (8)	5.5±0.5 (8)	P<0.001

### Experiment 4. Electrocardiogram (ECG), face temperature and electroencephalogram (EEG) recording during mental tasks

**Figure 1:**
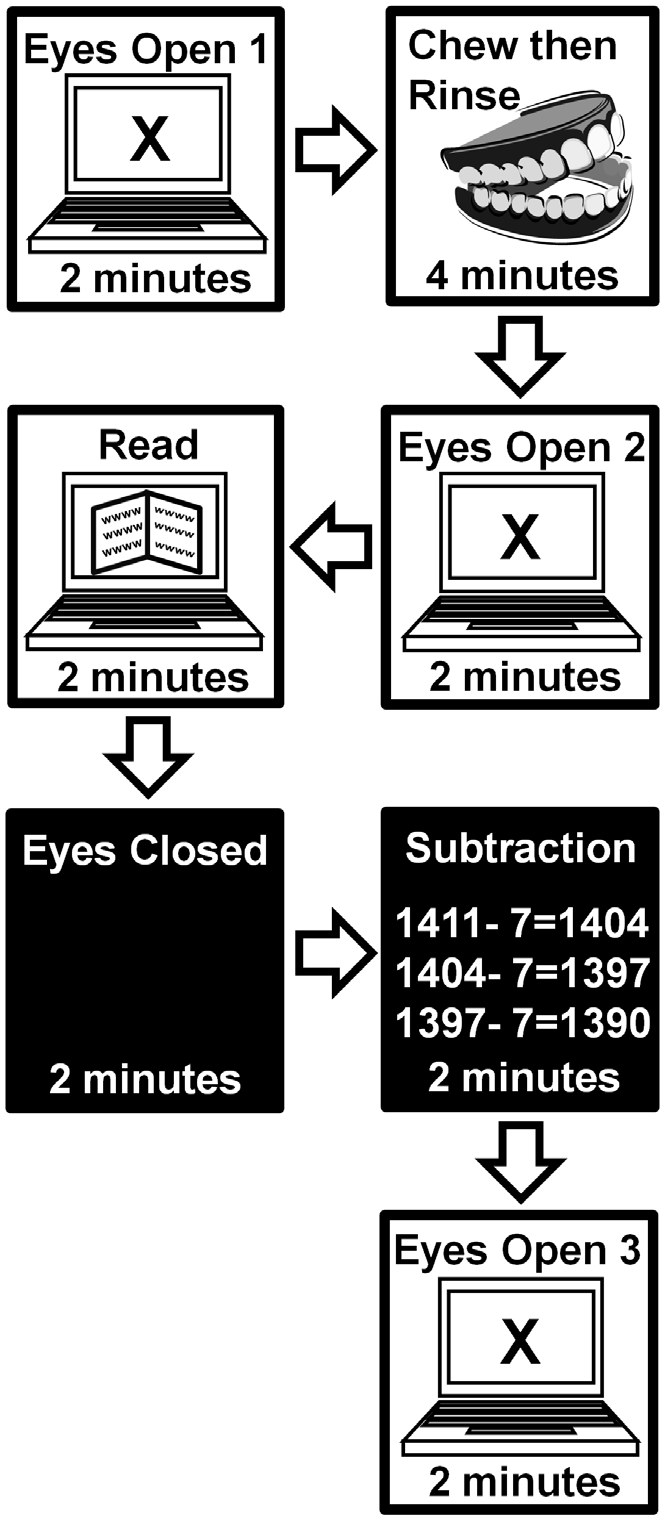
Diagram outlining the order of presentation and duration of mental tasks during the measurement of ECG, EEG and face temperature. Refer to text of material and methods, experiment 4 for specific details.

A diagram outlining the order of presentation and duration of mental tasks during the measurement of ECG, EEG and face temperature is presented in figure 1.

### ECG and face temperature

**Figure 2:**
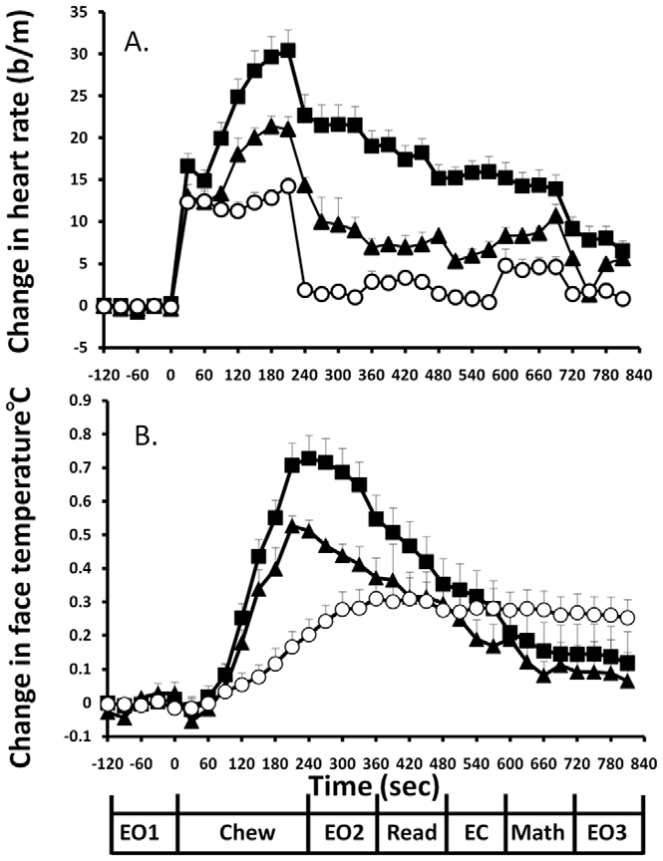
Time course of changes in heart rate and face temperature. The effect of chewing gum (open circle, n = 15) or chewing betel quid ((0.08 g/kg bwt (solid triangle, n = 3) and 0.14 g/kg bwt (solid square, n = 12)) on A.) change in mean basal heart rate (beats/minute) and B). change in mean face temperature (°C) presented at 30 second intervals during the performance of mental tasks. EO1 - Eyes open 1, Chew – Chewing treatment, expectorate and rinse mouth, EO2 - Eyes open 2 , Read – Reading text, EC – Eyes closed, Math – Eyes closed mental subtraction task, EO3 – Eyes open 3. Mean ± SEM. Mean basal heart rate for 12 subjects recorded in 2 minutes before chewing treatment was 73.7±1.5 beats per minute. Mean basal face temperature for 12 subjects recorded in 2 minutes before chewing treatment was 35.2±0.08°C.

The effect of chewing gum or chewing two doses of betel quid (0.08 g/kg bwt (n = 3) and 0.14 g/kg bwt (n = 12)) on mean heart rate (presented at 30 second intervals) while performing psychological tests is shown in figure 2. Statistical analysis was confined to comparisons between chewing gum and chewing the higher dose of betel quid (0.14 g/kg bwt) which produced peripheral physiological and presumably central effects for the duration of 12 minutes required to complete the mental tasks. Mean basal heart rate for 12 subjects recorded in 2 minutes before chewing treatment was 73.7±1.5 beats per minute. Mean basal face temperature for 12 subjects recorded in 2 minutes before chewing treatment was 35.2±0.08°C.

The physical act of chewing gum elevated mean heart rate by approximately 12 beats per minute for the duration of the chewing. Relative to Eyes open 1, reading elevated mean heart rate by 2 beats per minute for the duration of the procedure (P<0.05). Relative to Eyes open 1, eyes closed mental arithmetic elevated mean heart rate by approximately 5 beats per minute for the duration of the exercise (P<0.05). Chewing gum elevated face temperature by approximately 0.3°C for the duration of the experiment and face temperature appeared independent of mental exercise. Chewing betel quid dose dependently increased heart rate and face temperature. Chewing 0.14 g/kg bwt betel quid induced tachycardia above the physical effect of chewing by about 2 minutes after the onset of chewing. Tachycardia peaked at 30 beats per minute at 4 minutes after the onset of chewing. After expectoration and rinsing of the mouth with water, the mean increase in heart rate decreased to about 22 beats per minute. This tachycardia slowly decreased to about 7 beats per minute by the end of the measurement period. The ending of mental arithmetic task was coincident with a decrease in mean heart rate by about 6 beats per minute. Chewing 0.14 g/kg bwt betel quid induced an increase in face temperature above the physical effect of chewing by about 2 minutes after the onset of chewing. Mean increase in face temperature was maximal at 0.72°C about 4 minutes after the onset of chewing and decreased to 0.1°C above pre chewing temperature by the end of the experimental period. By about 10 minutes after the onset of chewing, face temperature was lower after chewing betel quid than after chewing gum. This probably is a consequence of face cooling after evaporation of sweat, the release of which was induced by betel quid.

### Electroencephalography

**Figure 3:**
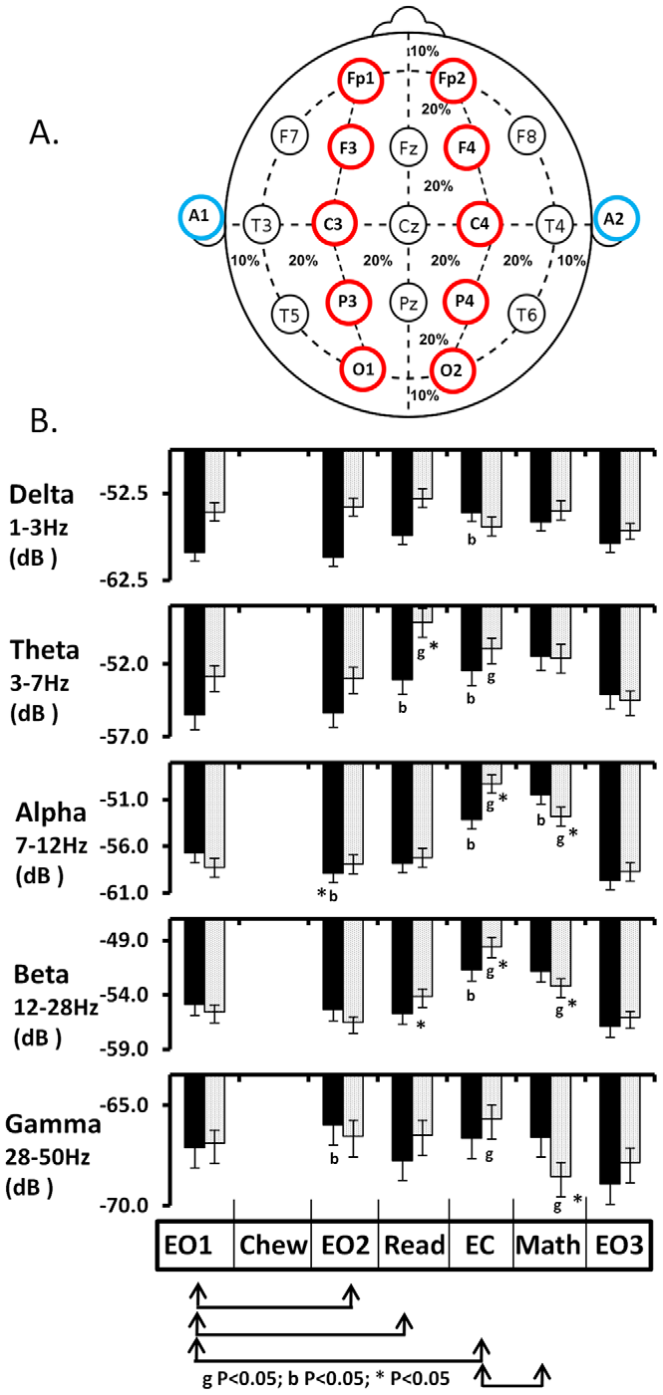
EEG electrode placement and global changes in EEG. A. EEG was recorded from 19 scalp electrodes (Fp1, Fp2, F7, F3, Fz, F4, F8, T3, C3, Cz, C4, T4, T5, P3, Pz, P4, T6, O1, O2) placed in accordance with the International 10–20 system, using an electrode cap with tin electrodes referenced to both ears (blue - A1, A2). Analysis is presented from 10 bilaterally symmetrical electrodes labeled in red. B. The effect of chewing gum (dotted histogram, n = 10) or chewing betel quid (0.14 g/kg bwt - solid histogram, n = 10) on change in mean global power (dB) of EEG spectral frequency bands. EO1 - Eyes open 1, Chew – Chewing treatment, expectorate and rinse mouth when EEG was obscured by EMG, EO2 - Eyes open 2, Read – Reading text, EC – Eyes closed, Math –Eyes closed mental subtraction task, EO3 – Eyes open 3. Mean ± SEM. Arrows indicate appropriate comparisons. g - Significantly different after chewing gum P<0.05; b - Significantly different after chewing betel quid P<0.05; * significantly different response between chewing gum and betel quid P<0.05.

EEG activity at scalp electrodes, when recorded with linked ear references as in this study, reflects the electrical activity of local and distant cerebral sources and inferences about topographic localizations of brain activity related to a particular mental task should be conservative [22]. EEG was recorded from 12 subjects but two were excluded from the EEG analyse because of the excessive influence of ECG signal or eye blink artifacts in either the gum or betel quid EEG recording. In each subject, EEG was recorded from 19 electrodes but because of computing restraints could only be analyzed from 10 symmetrically positioned electrodes. Fp1, Fp2, F3, F4, C3, C4, P3, P4, O1, and O2. See figure 3A. Spectral power (dB) in the delta, theta, alpha, beta and gamma frequencies at all electrodes was combined for global analysis of the effect of chewing gum or betel quid, on the six mental tasks (eyes open 1, eyes open 2, reading, eyes closed, eyes closed mathematics, eyes open 3). See Figure 3B. No significant difference was found between brain hemispheres for the effect of mental tasks or treatment for any frequency range. As such data from electrode pairs in prefrontal (Fp1, Fp2), frontal (F3, F4), parietal (C3,C4), posterior parietal (P3, P4) and occipital regions (O1, O2) were combined for 5 region repeated measures ANOVA analysis of topographic changes in spectral power on the six mental tasks.

#### Eyes open 1 - eyes open 2

**Figure 4:**
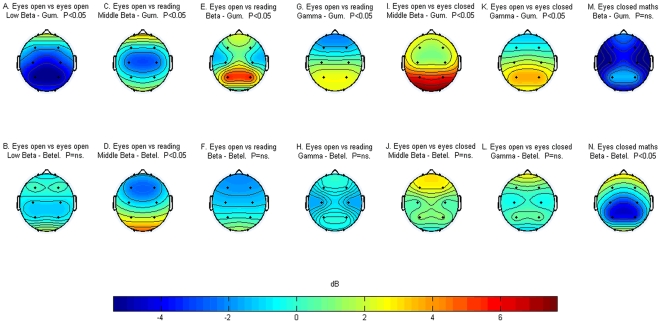
Topographic changes in EEG. Topographic representation from 10 subjects of regionally significant changes in mean spectral power from 10 electrodes (5 symmetrical electrode pairs) between mental tasks after chewing gum (A, C, E, G, I, K, M) or betel quid intoxication after chewing betel quid (B, D, F, H, J, L, N). (p<0.05, Tukey's post hoc corrections indicates frequency specific significant difference in magnitude of response to task between 5 brain regions. P = ns indicates no regional difference in response). Red indicates an increase in spectral power dB. Blue indicates a decrease in spectral power dB.

Chewing gum had very modest effects on the profile of spectra power of subjects with eyes fixated on a computer screen (comparisons of task 1 and task 2). Chewing gum significantly decreased the global power of low (P<0.005) and middle beta waves (P<0.015) but no significance regional differences in response to the task were apparent. (Figure 3B). Chewing gum significantly decreased the global power of low beta waves (P<0.005) with the greatest decrease being in the posterior parietal (P3, P4) and occipital regions (O1, O2) (P<0.05). (Figure 4A). Although no change was recorded in global alpha power after chewing gum, brain region analysis showed significant main effect but this did not retain significance after post hoc corrections. All other spectral bands, including the total beta wave band, were globally unchanged after chewing gum. Despite strong peripheral effects, betel quid intoxication had surprisingly modest effects on the profile of spectra power of subjects with eyes fixated on a computer screen (comparisons of task 1 and task 2). In contrast to chewing gum, global power of middle beta band was increased (P<0.008) but no significance regional differences in response to task 2 were apparent. Betel quid intoxication induced a significant global decrease in alpha power (P<0.01) but no significance regional differences in response to the task were apparent. Chewing betel quid induced a significant global increase in gamma power (P<0.016) but no significance regional differences in response to the task were recorded.

#### Eyes Open 1 – eyes open reading

Reading after chewing gum was associated with a marginally significant global increase in the spectral power of theta waves (Figure 3B) and topographically specific increase in beta and gamma power in posterior parietal region (P3,P4) (Figure 4E,G). These changes in spectral power to reading are generally consistent with previous EEG reports [24]. The topographic spectral profile recorded to reading was substantially modified by betel quid intoxication with attenuation of the increase in global theta power and a failure to develop topographic changes in beta or gamma power.

Specifically, after chewing gum, the performing of a reading task (task 3), relative to fixated eyes open (task 1), was associated with a global increase in the power of theta waves (P<0.039). The magnitude of the global spectral response to reading, relative to eyes open, after chewing gum was significantly different from the response after chewing betel quid for theta (P<0.0089), high alpha (P<0.026), beta (P<0.026), high beta (P<0.0024) frequencies. Although no change was recorded in global beta or gamma power after chewing gum, significant regional differences in response to the reading task were apparent for these frequencies. Beta power increased in the parietal region relative to other brain regions (P<0.05)(Figure 4E). Gamma power decreased in the pre frontal region (Fp1, Fp2) and increased in the occipital and posterior parietal regions (P<0.05)(Figure 3G). After chewing betel quid, the reading task relative to fixated eyes open was not associated with any significant global change in EEG power. In contrast to gum, regional changes in spectral power during betel quid intoxication failed to achieve significance in any frequency (Figure 4F,H).

#### Eyes open 1 - eyes closed

Closing eyes after chewing gum was associated with significant global increase in the spectral power of theta, alpha and beta waves (Figure 3B). Topographic specific increase in middle beta power was measured in occipital (O1, O2) and posterior parietal (P3, P4) regions (Figure 4I). Gamma power increased in the prefrontal region (Fp1, Fp2) and decreased in the posterior parietal regions (P3, P4) (Figure 3K). These changes in spectral power to reading are generally consistent with previous EEG reports [25], [26]. The global and topographic spectral profile recorded to reading was substantially modified by betel quid intoxication with attenuation of the increase in global alpha and beta spectral power, and region specific differences in the magnitude of the response of middle beta and gamma power failed to develop (Figure 4J,L).

After chewing gum, eyes closed (task 4) relative to fixated eyes open (task 1) was associated with a global increase in the power of theta waves (P<0.009), alpha waves (P<0.0001), low alpha waves (P<0.0001), high alpha waves (P<0.0001), beta waves (P<0.0001), low beta waves (P<0.0001), middle beta waves (P<0.0001), high beta waves (P<0.0001) and gamma waves (P<0.038). In comparisons between task 1 and task 4, regional differences in power were only observed in middle beta waves with the response from the occipital and posterior parietal cortex being significantly larger than prefrontal cortex (P<0.05)(Figure 4I) and in gamma waves with prefrontal power decreasing and occipital and post parietal power increasing (P<0.05)(Figure 4K). After chewing betel quid, eyes closed relative to fixated eyes open was associated with approximately 50% smaller but still significant global increase in power of delta waves (P<0.0029), theta waves (P<0.001), alpha waves (P<0.0001), low alpha waves (P<0.0001), high alpha waves (P<0.0001), beta waves (P<0.0001), low beta waves (P<0.0001), middle beta waves (P<0.0001). The spectral response to eyes closed relative to eyes open after chewing gum was significantly bigger than the response after chewing betel quid for alpha (P<0.0001), low alpha (P<0.05), high alpha (P<0.0001), beta (P<0.0004), low beta (P<0.0028), middle beta (P<0.0001), high beta (P<0.0001) frequencies. In contrast to chewing gum, betel quid induced a significant increase in the power of delta waves (P<0.0001) and no change was observed in the power of gamma waves (P<0.44). In comparisons between task 1 and task 4, no region specific differences in the magnitude of the response to closing eyes was observed after chewing betel quid.

#### Eyes closed resting – eyes closed mental subtraction

Eyes closed mental subtraction performed after chewing gum was associated with a significant global decrease in the spectral power of beta and gamma waves (Figure 3B) but regional differences in the magnitude of the responses did not attain statistical significance. A previous study found extensive augmentation of theta and gamma power in response to mental subtraction tasks but this study used an eyes open protocol to test mathematics [24] and as such the difference in experimental protocols makes comparison of results problematic. The spectral profile recorded to eyes closed mental subtraction was substantially modified by betel quid intoxication with attenuation of the decrease in global beta and gamma power (Figure 3B). In contrast to chewing gum, beta wave power was increased in prefrontal (Fp1, Fp2) and occipital (O1, O2) regions after betel quid intoxication (Figure 4N).

Specifically, after chewing gum, the performing of a mental subtraction task (task 5) relative to closed eyes (task 4) was associated with a global decrease in the power of high alpha waves (P<0.013), beta waves (P<0.0025), low beta waves (P<0.008), middle beta waves (P<0.0012), high beta waves (P<0.0013) and gamma waves (P<0.039). Regional differences in the magnitude of these responses did not attain statistical significance. In contrast after betel quid intoxication, the performing of a mental subtraction task relative to eyes closed was associated with no significant change in the spectral power of the majority of frequencies analyzed. A global increase in power was measured for alpha waves (P<0.0001) and high alpha waves (P<0.0001). Although no global changes were recorded in global beta power, beta power increased in prefrontal (Fp1, Fp2) and occipital (O1, O2) regions relative to other brain regions (P<0.05)(Figure 4N). The global spectral response to mental subtraction relative to eyes closed after chewing gum was significantly smaller than the response after chewing betel quid for alpha (P<0.0001), low alpha (P<0.022), high alpha (P<0.0001), beta (P<0.0001), low beta (P<0.0001), middle beta (P<0.0001), high beta (P<0.0001) and gamma (P<0.0001) frequencies.

Subjects perceived that relative to chewing gum, chewing betel quid tasted unpleasant, induced arousal and made concentration difficult. Subjective self assessment of the psychological effect of chewing treatment during the measurement of ECG, face temperature and EEG is presented in Table 4.

**Table 4:** Effect of chewing gum or betel quid on subjective self assessment after ECG, face temperature and EEG measurement.

Self assessment question	Chew Gum Mean ± sem (n)	Chew Betel Quid Mean ± sem (n)	Paired t-test significance
Taste. Bad = 1/Good = 10	6.8±0.5 (12)	3.8±0.5 (12)	P<0.001
Restful = 1/Excited = 10	3.8±0.4 (12)	6.2±0.5 (12)	P<0.001
Concentration. Easy = 1/Difficult = 10	4.7±0.6 (12)	6.1±0.5 (12)	P<0.05
Reading text. Not interesting = 1/Interesting = 10	5.8±0.7 (12)	5.2±0.4 (12)	ns
Memory. Poor = 1/Good = 10	4.9±0.5 (12)	4.6±0.6 (12)	ns
Maths difficulty. Easy = 1/Hard = 10	5.0±0.6 (12)	5.3±0.7 (12)	ns
Stanford sleep scale	1.2±0.2 (12)	1.1±0.2 (12)	ns

## Discussion

Betel quid intoxication can only be achieved after mastication of the betel quid. Mastication, by itself, increases cerebral blood flow [26] and increases heart rate. Our results demonstrate that chewing gum was an appropriate control behavior for the tachycardia associated with the act of chewing betel quid prior to the release and absorption of the pharmacologically active components of betel quid. However, chewing gum has been demonstrated to increase alertness, decrease reaction time, induce a more positive mood but to have no effect upon memory [27]. EEG changes associated with chewing gum are inconsistent, with effects ranging from no change to modest decreases in beta wave and increases in alpha power that may result from mastication and the flavor of the gum being chewed [28], [29]. In the current study the effect of chewing gum similarly appears to have been limited to a decrease in the global power of low beta waves that was stronger in occipital regions and a borderline significant increase in alpha power in the prefrontal regions. As such chewing gum does influence cognitive processes, by unknown mechanisms, that are currently the focus of investigation [27]. In the context of this experimental series chewing gum is not a totally benign control but the changes are of a more subtle physiologically nature than the changes associated with betel quid intoxication.

It should be noted that although chewing gum was an adequate masticatory and cardiac control for the act of chewing betel quid it was not an appropriate psychological control for chewing betel quid. Subjects were not blind to the chewing treatment being tested. Although the psychoactive components of betel quid are not yet firmly established, future studies will have to develop a control condition that more closely resembles the characteristic texture, flavor and smell of betel quid so as to enable the blind testing of subjects.

### Prospective Time

The perception of time is a complex but fundamental aspect of human cognition. In addition to the prospective or retrospective nature of the specific time interval of interest [30], it is well recognized that motivational states and levels of attention influence our perception of the passage of time [30]. Accordingly models of cognitive time perception attempt to incorporate the influence of attention and arousal on the activity of temporal pacemaker and temporal buffers [31], the temporal properties of memory [32] and the duration of the temporal interval under investigation [30]. In naïve subjects, betel quid altered brain EEG activity, the subjects perceived level of arousal and induced an over estimate of the passage of a prospective 60 second time interval. The experimental protocol employed did not prevent subjects from using strategies to estimate the elapsed temporal interval, and some subjects reported that they employed some form of counting strategy. As such the effect of betel quid cannot be attributed solely to an effect upon an internal clock but must also be considered to have a component effecting cognitive processes employed in estimating a prospectively estimated interval.

### Working Memory

Betel quid had an effect not different from chewing gum on the digit span of numbers or non-vowel Roman alphabet characters. These data are consistent with the previous study in habitual users [5]. Digit span is not a comprehensive test of memory but it is a reliable indicator of a component of working memory [33]. It was surprising that even though the subjects consistently reported that they perceived betel quid to make thinking difficult or that they were not confident in their mental abilities after chewing betel quid, the subjects digit span was unaffected by chewing betel quid. In contrast, previous human studies document that pure arecoline, the presumed principal component of betel quid [6], [10], acting together with a choline supplement enhanced memory in healthy humans [34], by itself enhanced working memory in Alzheimer's patients [35], [36] and enhanced spatial memory performance in animal models of Alzheimer's disease [37], as do chemical derivatives of arecoline [38]. From a pharmacological perspective, the lack of effect of betel quid on character digit span, which was significantly less than numerical digit span, was surprising.

This suggests that pharmacologically active components of betel quid may be two or more compounds that act on different brain regions to give a false perceived impression of mental performance. Alternatively mental function is actually more difficult because of the opposing but balancing effect of memory enhancing and inhibiting compounds released from betel quid.

### Two choice reaction tests

Two previous studies have shown that chewing betel quid in habitual users had no effect upon simple reaction time [5], [21]. In the computerized two choice paradigm used in the current study, relative to chewing gum, chewing betel quid had no consistent effect upon two choice reaction time or the accuracy of the response in naïve subjects. Although subjects reported being more alert after chewing betel quid there was no relationship between perceived taste of betel quid or perceived effects of betel quid and performance. This result is inconsistent with a previous report which suggested that chewing betel quid by habitual chewers decreased reaction time in a three choice test [21]. Our results suggest that in naïve, healthy, non sleep deprived subjects, betel quid intoxication acts to facilitate a psychological arousal but this arousal is not reflected in an increase or a decrease in planning or visual-motor performance of simple tasks. Subjects perceived that concentration was more difficult after chewing betel quid but performance, when compared against chewing gum, was unchanged. In Taiwan, betel quid is frequently consumed day and night by drivers to maintain alertness. Our research suggests that continued consumption of betel quid in non-sleep deprived subjects may heightened the perception of alertness but this is unlikely to improve function in brain regions that attend to visual-motor co-ordination as is widely believed by the general community. In tired (sleep deprived) subjects betel quid is generally considered by users to have an arousing effect but no tests have yet been performed to determine if the perceived arousal is reflected in enhanced cognitive or motor performance.

### Electroencephalography

EEG is mainly derived from the summation of ongoing excitatory and inhibitory post synaptic potentials. Changes in EEG spectral power, without the use of time-locked averaging of repeated tasks or more complicated procedures, were historically used to demonstrate that specific cognitive behaviors are associated with global and/or regional changes in the power of spectral frequency bands. Although theories about the underlying mechanisms continue to evolve [25], the closing of eyes to visual input in a resting state has long been known to be associated with an increase in absolute spectral power of delta, theta, alpha and beta frequencies [24], [25]. Other EEG studies have demonstrated regional increases in theta power and wide spread increases in gamma power for reading tasks relative to the eyes open fixated control condition [23]. Mental subtraction in an eyes open state has been shown to induce local and widespread increase in theta and gamma power [23]. In the current experimental series, the spectral profiles associated with the performance of the mental tasks after chewing gum are generally consistent with previous literature and differences may result from variations in task, language and analytical procedures.

Betel quid intoxication induced large cardiac, hemodynamic and thermal effects on the body but relative to chewing gum, betel quid intoxication with open eyes fixated on a computer screen had very modest effects on EEG profile of spectra power of subjects. Changes were largely limited to a small decrease alpha wave power. However, betel quid intoxication clearly attenuated the global increase in alpha, beta and gamma power during periods of free thinking when eyes were closed and significantly altered the global and topographic spectral profiles of EEG when subjects were performing complex mental tasks such as mathematics and reading.

The experimental design employed self testing protocols and post experimental questioning of subjects was only used to confirm if the subjects complied with the performance of the mental tasks and could not be used to assess the level of compliance or if there was a difference in the cognitive performance of the subjects doing mental tasks after chewing gum or after betel quid intoxication. Subjects reported no difference in subjective measures of the interest of the reading passages or the number of self reported mistakes for the mathematics task between chewing treatments. This, together with reversible increase and decrease in heart rate recorded during the mathematics task when chewing gum, that was visible to a lesser extent after chewing betel quid, suggests that subjects were diligent in their attempts to comply with the performance of the mental tasks. Subjects reported that concentration was difficult when performing mental tasks after chewing betel quid and the different spectral profiles observed between the tasks of reading, eyes closed and mental mathematics after chewing gum or betel quid imply that the EEG may have portrayed this mental difficulty. Clearly future experiments need to address the question of whether the altered EEG spectral profiles recorded from cognitive tasks during betel quid intoxication, thought to in part reflect changes in regional cerebral metabolism and blood flow [39], do actually reflect altered or compromised performance during cognitively demanding tasks. The existence of such a relationship may have practical implications for the performance of complex tasks, such as driving, while under the influence of betel quid intoxication.

### Conclusions

Betel quid is a socializing drug shared at meetings of people in certain communities in much the same manner as tobacco was shared in the 1950 s and 60 s in western society. Betel quid intoxication is characterized by its rapid onset and short duration. Betel quid is similar to other socially sanctioned drugs of addiction such as tobacco, coffee and alcohol in that naïve subjects perceive the initial exposure to be unpalatable but the effect of the drug coupled with a level of social acceptance combine to determine that some individuals will become repeat users and a subset of these will become addicted. Betel quid contains a complex mixture of compounds, the CNS pharmacological actions of which have yet to be exactingly characterized. In naïve, non-sleep deprived subjects, gross behavioral indices of working memory, simple visual-motor performance were not affected by betel quid intoxication, but the psychological aspects of perception such as slowing of prospective time and perceived levels of arousal and perceived ability to think were strongly influence by betel quid intoxication. The differential effects of betel quid intoxication on EEG profiles, behavioral and psychological performance suggests that betel quid intoxication does not influence cerebral functions via a global mechanism but alters the function of selective populations of neurons that subtend to specific human perceptions by an as yet undetermined chemical nature. The functional specificity of the action of betel quid intoxication on human behavior suggests that the central effects of betel quid intoxication may be amenable to investigation with fMRI.

## Materials and Methods

### Subjects

The nature, purpose and risks of the study were explained to each subject before written informed consent was obtained. The experimental protocol conformed to the standards set by the Declaration of Helsinki and the study protocol (#200907) was approved by the ethics review committee of National Dong Hwa Univerisity on 6^th^ October, 2009.

Male students and teachers were recruited from Dong Hwa University campus. The study was limited to males because university educated females considered chewing betel quid to be an unacceptable behavior and could not be recruited for the experiment. Naïve subjects were recruited for study so as to avoid the effects of physiological adaptation that may occur in habitual chewers. Some subjects had very limited experience of chewing betel quid at least two years prior to the study and none had ever been regular users. Subjects were selected after an initial screening to determine that they had no history of psychiatric illness, heart disease, chronic medical condition or history of betel quid consumption within the last two years. Thirty four men were solicited for the study, three were rejected on the basis of a self disclosed, preexisting history of heart irregularities. Thirty-one men aged 20–54 years old, (mean = 33, SD = 13) participated in this study. All tasks were performed while subjects were seated comfortably at a desk facing a computer screen. Eight subjects participated in the time interval estimation task (Experiment 1). The same eight subjects participated in the working memory task (Experiment 2). Eight different subjects participated in the computerized two choice test of reaction time task (Experiment 3). Fifteen different subjects participated in the bio-electric measurement and mental task (Experiment 4). All participants were non-smokers. The majority of subjects received $NT 500 compensation for participation, after the study was complete. Participants were instructed not to drink tea or coffee in the 2 hours before testing. In each experiment Wrigley's Doublemint ® chewing gum was used as the masticatory control for chewing betel quid.

### Experimental procedures

#### Experiments 1 and 2

Eight subjects (22–55 years old, mean = 36, SD = 14) participated in this study. Two subjects had very limited experience of chewing betel quid at least two years prior to the study and neither had ever been a regular user. Experiments 1 & 2 took place between 12 pm and 2 pm before lunch. Experiments 1 and experiments 2 were performed sequentially in the same experimental session. On the first experimental task session, a test session was performed to familiarize the subjects to the time interval assessment task, memory tasks and the subjective self-assessments. Subjects were then randomly assigned to a treatment type 1) chew betel quid for 2 minutes or 2) chew gum for 2 minutes. Each subject completed both treatments separated by a week. Subjects were instructed not to swallow any juices produced from chewing. After 2 minutes chewing the subjects expectorated and rinsed their mouth with water and wiped their face. A period of 1 minute was allowed for the seated subject to relax before testing commenced. Experiments 1 & 2 were completed within 10 minutes of expectorating the chewed treatments. After the completion of experiment 2 subjects were asked for a subjective assessment of the following questions.

Did the treatment taste Bad – Good.

Did the treatment make it Difficult to concentrate – Easy to concentrate.

Did the treatment make your face feel hot. Yes-No.

Did the treatment make your heart beat faster. Yes-No.

Did you use a counting strategy to estimate the passage of time for 5 seconds or 1 minute.

### Experiment 1. Prospective time interval estimation after chewing betel quid or gum or sham chewing

Seated subjects were given a digital stop watch (Seiko 9N8678, Japan) which they operated but could not see the watch face and were asked to estimate the passage of 5 seconds from any time of their deciding. This time was recorded by the observer. Subjects were then asked to repeat the procedure and estimate the passage of 60 seconds. One week after the completion of the chewing gum and betel quid treatments subjects were recalled to perform and additional prospective time interval estimation task to determine if sham chewing had an effect upon time interval assessment. At the 3rd measurement of the prospective time interval estimation task, the seated subjects were asked to imagine they were chewing for 2 minutes and expectorating but were given nothing to chew or drink. Subjects then estimated the passage of 5 sec and 60 seconds. Post task questioning revealed that most subjects used some strategy to estimate the time interval.

### Experiment 2. Numeral and character digit span task when chewing betel quid or gum

Numerals. Head phones were used to acoustically present pre-recorded series of 4–11 digit numbers in ascending and then descending sequence. A female voice introduced each sequence and announced numbers every 30 seconds. A tone immediately after the last number spoken indicated when subjects should write down the sequence. Characters. A computer program was used to randomly present a series of 4–11 non-vowel, roman alphabet characters for 5 seconds on a computer screen. Seated subjects were instructed to write down the numbers immediately upon their disappearance from the screen. There was a 30 second interval between the presentation of characters.

### Experiment 3. Computerized 2 choice reaction task when chewing betel quid or gum

Eight subjects (24–53 years old, mean = 38, SD = 11) participated in this study. Two subjects had very limited experience of chewing betel quid at least two years prior to the study and neither had ever been a regular user. Experiment 3 took place at 3–4 pm after lunch. Subjects completed a computerized fixed interval, continuous 2 choice test of visual-motor performance of 9.5 minutes in length. This test has no language demands and requires no left-right discrimination. The black target box was presented at the midline of a light computer screen for 200 milliseconds every 2 seconds on 243 occasions. The position of the target box was unpredictably varied between the upper half of the computer screen (correct response) and the lower half of the screen (incorrect response). The target was in the correct (upper) field on 99 of these presentations. Subjects rested their finger on the computer space bar key and were instructed to respond by pressing the computer space bar when the target box appeared in the upper field of the computer screen as quickly and accurately as they could. Subjects were not told that reaction time was being recorded as part of their responses to the test. Prior to each experimental 2 choice test, each subject was given a 2 minute introductory familiarization task to practice the target presentation and response protocol.

The effect of two chewing treatments, chew gum for 2 minutes or chew betel quid (0.08 g/kg body weight (bwt)) for 2 minutes on 2 choice visual-motor responses were examined in eight subjects. The order of betel quid and gum chewing treatment presentations was counterbalanced with half of the participants performing first under the betel quid treatment and half under the gum treatment. Subjects were instructed not to swallow any juices produced from chewing. After 2 minutes chewing the subjects expectorated and rinsed their mouth with water and wiped their face. A period of up to 1 minute was allowed for the seated subject to relax before the 2 choice visual-motor test commenced. An interval of one hour separated the tests of the two chewing treatments. This time interval was chosen because 1) it was sufficient for the effects of betel quid or chewing gum to have dissipated 2) it minimized any differences in reaction time that may occur when testing was performed at different times of day or variation in motivational states between testing on different days and 3) it minimized the time the subjects needed to devote to testing.

After the completion of each 2 choice test each subject gave a subjective assessment using visual analogue scale of the following questions.

Did the treatment make your face feel hot. Yes-No.

Did the treatment make your heart beat faster. Yes-No.

Did the treatment taste Bad (1) – Good (10)

Did the treatment make you feel Restful (1) – Excited (10)

Did the treatment make it Difficult to concentrate (1) – Easy to concentrate (10)

### Experiment 4. ECG, EEG, facial temperature recording during mental tasks after chewing betel quid or gum

Fifteen subjects (20–53 years old, mean = 29, SD = 11) participated in this study. One subject had very limited experience of chewing betel quid at least two years prior to the study and had never been a regular user. At the subject's first experimental session, height, body mass and cuff blood pressure (Omron Hem 711AC) and questionnaires about occupation and level of exercise were completed. A familiarization task was performed to introduce each subject to the mathematical task and determine the speed of presentation of characters in the reading task. This familiarization task proceeded each experimental session. EEG, ECG and facial temperature were recorded simultaneously with the performance of each mental task.

Subjects were randomly assigned to an initial chewing treatment. Three chewing treatments were examined. 1) Chewing betel quid (0.08 g/kg bwt) in the morning between 9–11 hours, N = 3 subjects; 2) Chewing betel quid (0.14 g/kg bwt) in the morning between 9–11 hours, N = 12 subjects; 3) Chewing 4 pieces of gum in the morning between 9–11 hours (n = 15 subjects). Each subject chewed gum and betel quid once. The time interval between chewing treatments was 2–7 days. Three subjects were chosen to consume a lower dose of betel quid because a previous review had implied that betel quid may not have a dose dependent effect on heart rate [5].

### Experimental protocol

Subjects were seated comfortably in front of a computer in a chair that supported the subject's arms and were asked to remain as motionless as possible. Feet rested stationary on an electrically insulated floor. Chair height was adjusted for maximum comfort of viewing the computer screen. A modified Stanford Sleepiness Scale (SSS) self assessment containing ratings 1) alert & wide awake, at your mental peak; 2) able to concentrate but not a mental peak; 3) hard to concentrate , a bit tired; 4) sleepy, require effort to concentrate; and subjective assessment of hours of sleep in the previous night were administered before every recording session. After connection of the EEG, ECG and face temperature sensor the subjects completed the following experimental task protocol which consisted of a control measurement period, a period of chewing then expectorating and rinsing mouth, followed by five sequential presented mental tasks. Each mental task was separated by a 5–10 second interval where the subjects were given instructions and commenced the task. A diagram outlining the order of presentation and duration of mental tasks during the measurement of ECG, EEG and face temperature is presented in Figure 1.

#### Visual fixation 1 - task 1

Subjects were asked to relax and visually fixate for 2 minutes a stationary X, 1 cm tall, presented on the center of the computer screen.

#### Chewing treatments

Betel quid. Subjects were asked to chew 1 betel quid for 2 minutes, expectorate, chew a second betel quid for 1.5 minutes, expectorate, rinse mouth with water, expectorate and wipe face. Total time 4 minutes. Chewing gum. Subjects were asked to chew two pieces of commercial gum for 2 minutes, then to add two more pieces of gum and continue to chew for 1.5 minutes, expectorate, rinse mouth with water, expectorate and wipe face. Total time 4 minutes.

#### Visual fixation 2 - task 2

Subjects were asked to relax and visually fixate for 2 minutes a stationary X, 1 cm tall, presented on the center of the computer screen.

#### Reading - task 3

Subjects were asked to silently read a Chinese text for 2 minutes. The text scrolled automatically up the page at a speed that was predetermined to be the most comfortable for the subject. To minimize head and eye movements the text was only one character wide and was presented in the middle of the screen. Subjects read one of four texts about travel in Germany, Poland, Tunisia and Brazil. Texts were initially randomly assigned to a subject thereafter texts were allocated to ensure a balanced design for each chewing treatment.

#### Eyes closed – task 4

Subjects were asked to closed their eyes and relax for 2 minutes.

#### Mathematics – task 5

Subjects with eyes closed were given a 4 digit number 1,211, 1,311, 1411, 1511 and asked to mentally subtract 7 repeatedly for 2 minutes. Numbers were initially randomly assigned to a subject thereafter numbers were allocated to ensure a balanced design for each chewing treatment.

#### Visual fixation 3 - task 6

Subjects were asked to relax and visually fixate for 2 minutes a stationary X, 1 cm tall, presented on the center of the computer screen.

### Post experimental subjective self assessment

After the completion of each chewing treatment protocol subjects gave a subjective assessment using visual analogue scale of the following questions.

Repeat of the modified Stanford sleep scale.

Did the treatment taste Bad (1) – Good (10)

Did the treatment make you feel Restful (1) – Excited (10)

Did the treatment make it Difficult to concentrate (1) – Easy to concentrate (10)

Was the reading passage interesting 1–10

Can you remember the passage Poorly (1) – Well (10)

Some questions specific to each reading text to confirm that the passage was read.

Was the subtraction task Difficult (1) – Easy (10).

What numbers did you get stuck on.

Subjects answered questions immediately after completing the last mental task.

### Bio-electric recordings

The EEG was recorded from 19 scalp electrodes (Fp1, Fp2, F7, F3, Fz, F4, F8, T3, C3, Cz, C4, T4, T5, P3, Pz, P4, T6, O1, O2) placed in accordance with the International 10–20 system, using an electrode cap with tin electrodes referenced to both ears (Electro-cap International Inc, Ohio, USA). See Figure 2A. Electrode gel (Electro-cap International Inc) was added to each electrode to improve contact. EEG was digitized using a 16 bit A/D converter at a sampling rate of 512 points per channel per second, with a high frequency filter of 70 Hz and a low frequency filter of 0.1 Hz using a commercial acquisition system (NP-Q10/20 Neuropulse Systems, Colorado, USA). Data were reviewed off line for the manual removal of eye movement and swallowing artifacts. The first 5–10 seconds of each 2 minute EEG recording were also removed from the analysis to avoid evoked responses to the eyes-close/open signal or other commands. Absolute EEG power (intensity of energy in a frequency band in the delta (1.0–3.0 Hz), theta (3–7 Hz), alpha (7–12 Hz), low alpha (7–9 Hz), high alpha (9–12 Hz), beta (12–28 Hz), low beta (12–16 Hz), middle beta (16–20 Hz), high beta (20–28 Hz) and gamma (28–50 Hz) bands of the entire measurement period were calculated using a fast Fourier transformation and normalized by time. Results were presented as dB (20*log(mV^2^) [40].

ECG was recorded from disposable adhesive gel electrodes positioned around heart. ECG was measured using Biopac MP-35 (Biopac Systems, California, USA) with low frequency filter of 0.2 Hz and high frequency filter of 50 Hz low at a sampling rate of 200 Hz. Facial temperature of the left cheek from the zygomaticus muscle above cheek bone was measured using a thermistor probe connected to Biopac MP-35 with low frequency filter of 0.2 Hz and high frequency filter of 50 Hz at a sampling rate of 10 Hz. Data was stored on a dedicated windows XP laptop PC for post experimental analysis. Room temperature was maintained at 28°C using a thermostatically controlled air conditioner.

### Statistical analyses

Results were analyzed using Statistical Analysis System (SAS v8.1). Experiment 1 was analyzed by repeated measure ANOVA on the omnibus difference across 3 conditions. Experiment 2 was analyzed by paired t-test. Experiment 3 was analyzed by paired t-test. Experiment 4. EEG was analyzed by repeated measures ANOVA with 3 within factors, 2 treatment conditions (gum and betel quid) 6 mental tasks and 5 brain regions. Subsequent analysis of tasks of interest employed one within factor repeated measures analysis with Tukey's post hoc corrections for mental tasks of interest (eg., Eyes open 1, Eyes open 2, Eyes open 3; Eyes open 1, Eyes closed, Eyes open 3; response across brain regions) for each frequency range. Statistically significant regional differences were presented graphically using Matlab. Global effects were determined by combining data for specific frequencies from all electrodes and paired t-tests were employed to detect significant differences between mental tasks of interest (eg., Eyes open 1 vs. Eyes closed, Eyes open 1 vs. Read, Eyes closed vs. Maths, change in response to betel quid vs. change in response to gum). Bonferoni method controlled overall experiment wise type 1 error rate. ECG. Change in heart rate was analyzed by the single sample t-test of whether the mean differed significantly from zero. Bonferoni method controlled overall experiment wise type 1 error rate. Subjective estimates were analyzed by paired t-test. The level of statistical significance was set at P<0.05. Data are expressed as mean ± SEM.
